# Emergence of Lyme Disease on Treeless Islands, Scotland, United Kingdom

**DOI:** 10.3201/eid2702.203862

**Published:** 2021-02

**Authors:** Caroline Millins, Walter Leo, Isabell MacInnes, Johanne Ferguson, Graham Charlesworth, Donald Nayar, Reece Davison, Jonathan Yardley, Elizabeth Kilbride, Selene Huntley, Lucy Gilbert, Mafalda Viana, Paul Johnson, Roman Biek

**Affiliations:** University of Liverpool, Liverpool, UK (C. Millins);; University of Glasgow, Glasgow, Scotland, UK (C. Millins, W. Leo, D. Nayar, R. Davison, J. Yardley, L. Gilbert, M. Viana, P. Johnson, E. Kilbride, R. Biek);; National Health Service Western Isles, Benbecula, Scotland, UK (I. MacInnes);; Scottish Natural Heritage, South Uist, Scotland, UK (J. Ferguson);; Southern Isles Veterinary Practice, Benbecula (G. Charlesworth);; Heb Insights, North Uist, Scotland, UK (S. Huntley)

**Keywords:** Lyme disease, treeless habitats, *Ixodes ricinus*, peridomestic, tick bite exposure, deer, emergence, tick-borne infections, bacteria, ticks, vector-borne infections, zoonoses, One Health, bacterial zoonoses, Scotland, United Kingdom, *Borrelia burgdorferi*

## Abstract

Lyme disease is usually associated with forested habitats but has recently emerged on treeless islands in the Western Isles of Scotland. The environmental and human components of Lyme disease risk in open habitats remain unknown. We quantified the environmental hazard and risk factors for human tick bite exposure among treeless islands with low and high Lyme disease incidence in the Western Isles. We found a higher prevalence of *Borrelia burgdorferi* sensu lato–infected ticks on high-incidence than on low-incidence islands (6.4% vs. 0.7%); we also found that residents of high-incidence islands reported increased tick bite exposure. Most tick bites (72.7%) occurred <1 km from the home, including many in home gardens. Residents of high Lyme disease incidence islands reported increasing problems with ticks; many suggested changing deer distribution as a potential driver. We highlight the benefits of an integrated approach in understanding the factors that contribute to Lyme disease emergence.

To optimize public health responses to vectorborne disease emergence, knowledge of the factors affecting the density of infected vectors in different habitats, human interactions with the environment that lead to vector exposure, and how these factors affect disease incidence are essential. Lyme disease, caused by infection with the bacterium *Borrelia burgdorferi*, is the most commonly reported vectorborne zoonotic disease in Europe and North America (*1*,*2*). Higher densities of infected tick vectors (i.e., environmental hazard) and Lyme disease incidence are associated with wooded habitats (*3*–*5*). However, the recent emergence of Lyme disease on treeless islands in Scotland (*6*), United Kingdom, has challenged the current understanding of the relationship between habitat and Lyme disease. 

Lyme disease is an emerging zoonosis in the United Kingdom; the highest incidence is in the Highland region of Scotland (*7*,*8*). In the United Kingdom, Lyme disease surveillance is based on laboratory confirmed cases, following the best practice guidelines for serologic diagnosis published by the National Institute for Health and Care Excellence (*9*–*11*). This surveillance shows that some islands in the Highland region that lack woodland coverage have a Lyme disease incidence ≈40 times the national average (119 vs. 3.2 cases/100,000 persons per year) (*6*). These islands have had a higher Lyme disease incidence since at least 2010; other nearby, ecologically similar islands have a much lower incidence of 8.3 cases/100,000 persons (*6*). These islands also have a higher incidence of Lyme disease diagnoses made on the basis of an erythema migrans rash (*6*,*11*). Knowledge of the factors affecting the density of infected ticks in the environment, how persons interact with the environment and are exposed to tick bites, and possible drivers of emergence is urgently needed to examine, predict, and mitigate Lyme disease emergence in treeless habitats.

Evidence suggests that Lyme disease hazard (measured as the density of infected ticks) is lower in treeless habitats than in wooded areas; however, much about this relationship remains unknown (*12**–**18*). Many experts consider woodlands to be the optimal habitat for the Ixodid tick vector because of the humid microclimate, which improves off-host tick survival and the density of potential hosts for blood meals (*12*,*13*). Some studies have found lower tick densities in grassland than in nearby woodland habitats, prompting researchers to theorize that grassland might act as a sink for tick populations (*14*–*16*). Furthermore, many studies have found the density of the *Ixodes ricinus* tick, the main vector of Lyme disease in Europe, to be much lower in treeless habitats than woodlands (*17*). For example, surveys of open habitats in northern Spain found no questing *I. ricinus* ticks (*18*). In the United Kingdom, most studies have found relatively low tick densities in meadows (*19*), open hillside (*20*,*21*), and heather moorland (*22*,*23*).

The environmental hazard is linked to Lyme disease incidence through human interactions with the environment and exposure to infected tick bites (*24*). For example, a person’s activities, knowledge of and attitude toward tickborne disease, and preventative behaviors will affect that person’s risk for tick bites (*24*,*25*). Analysis of where people are exposed to tick bites and risk factors for tick bite exposure can be used to guide preventive public health interventions (*26*).

In the absence of longitudinal environmental data in treeless areas, alternative approaches are needed to assess trends in tick population abundance and distribution. Tick populations in treeless habitats are affected by many of the same environmental drivers as those in forested areas, such as changes in climate, land management, and host density, especially deer populations (*27*–*30*). Surveys of local communities can provide information on whether the tick hazard is perceived to have changed over time. Responses might also suggest environmental factors associated with these changes (*31*). 

To identify possible causes of Lyme disease emergence in treeless habitats, we assessed factors influencing tick density and prevalence of *B. burgdorferi*–infected ticks; geographic, demographic, and behavioral factors associated with human tick bite exposure; and community recollections of tick distribution and numbers over time. We used treeless islands with high and low Lyme disease incidence in the Western Isles in Scotland, United Kingdom, as our study system.

## Methods

### Study Location and Site Selection

**Figure 1:**
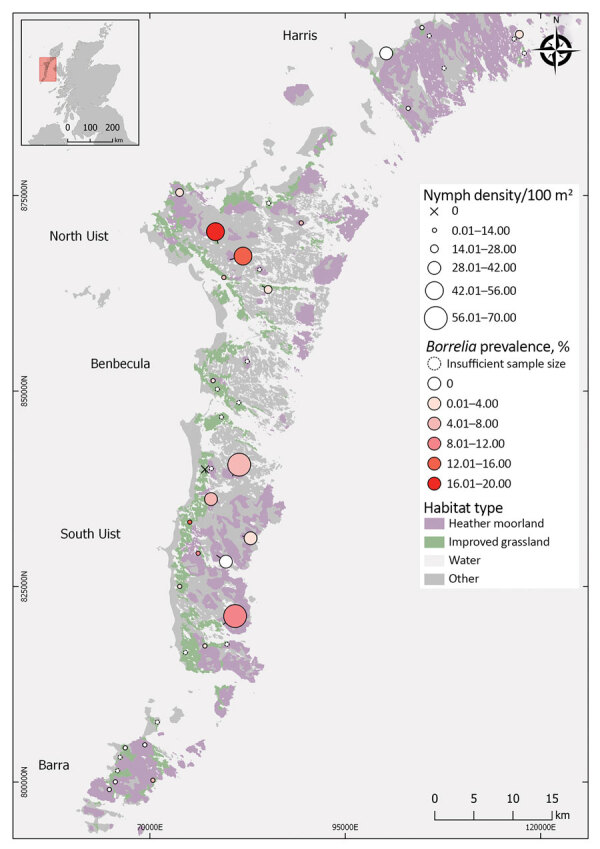
Tick collection sites for study on Lyme disease hazard, Western Isles, Scotland, UK, 2018–2019. Prevalence was not estimated at sites where <50 ticks were collected. Circle size indicates questing tick density. Circle color indicates *Borrelia burgdorferi* sensu lato prevalence. X indicates sites at which no ticks were detected.

We classified each island as having a low or high Lyme disease incidence based on Lyme disease surveillance data (*6*). We compared the environmental hazard between 26 sites on islands with high Lyme disease incidence (North Uist, South Uist, and Benbecula) and 16 sites on islands with low incidence (Harris and Barra). We selected sites belonging to 2 dominant habitat types: improved grassland (mesotrophic grasslands, often used for livestock grazing) and heather moorland (a mixture of wet heathland and western blanket bog) (*32*). We used a spatially stratified sampling design and the random selection tool in QGIS (QGIS Development Team, https://www.qgis.org) to select sites (Figure 1). The vertebrate community of the Western Isles includes large ungulates, such as wild red deer (*Cervus elaphus*), farmed sheep, and cattle, all of which can maintain *I. ricinus* tick populations. The islands also have several *B. burgdorferi* sensu lato transmission hosts, including brown rats (*Rattus norvegicus*), Eurasian pygmy shrews (*Sorex minutus*), wood mice (*Apodemus sylvaticus*), hedgehogs (*Erinaceus europaeus*), field voles (*Microtus agrestis*), and certain species of passerine birds (*33*).

On islands where Lyme disease incidence is high (high-incidence islands), we also selected sites belonging to 3 additional habitats. We chose 8 sites in machair and 13 sites in bog and peatland habitats using the same stratified sampling approach. Machair is a sandy grassland along ocean coastline often used for grazing or cultivation (*32*). We also chose 12 sites in gardens that were randomly selected within each sector (Appendix Figure 1). Sampling was carried out during the peak questing period for *I. ricinus* ticks. We conducted sampling during April 19–June 5, 2018. To strengthen the comparison of tick infection prevalence, we sampled additional sites in low Lyme disease incidence (low-incidence) areas during May 17–June 22, 2019.

### Tick Collection

To estimate the density of questing *I. ricinus* ticks, we sampled from 20 randomized 10 m transects at each site. Transects were 30–50 m apart, or 20–30 m apart in gardens. We measured vegetation height and density, temperature, and humidity at the starting point of each transect (*34*). We dragged a 1 m^2^ white woolen blanket across the surface of the vegetation for 10 m. We collected questing nymphs on the blanket, counted them, and placed them in 100% ethanol. To increase the sample size, we carried out continuous blanket dragging for <2 person-hours at each site.

### Screening of *I. ricinus* Ticks for *B. burgdorferi* s.l. and Genospecies Identification

Our pilot study on South Uist in 2017 estimated that 6.6% of *I. ricinus* nymphs were infected with *B. burgdorferi*; we used this preliminary prevalence to estimate a target sample size of 50 nymphs/site (C. Millins, unpub. data). We used an ammonia hydroxide technique (*35*) to extract approximately 50 *I. ricinus* questing nymphs collected by blanket dragging at each site. We tested the ticks for *B. burgdorferi* s.l. infection using a nested PCR specific to the flagellin gene (*36*) with sequencing of the product to identify the genospecies.

### Geographic Locations of Human Tick Bite Exposure, Factors Associated with Tick Bite Risk, and Perceptions of Tick Problems Over Time

We invited residents to complete a questionnaire about tick bite exposure. We used the survey to collect data about differences in tick bite exposure between islands with high and low Lyme disease incidence, habitat types where tick bites occurred, the distance of tick bites from the home address, and social and behavioral factors associated with exposure to tick bites. Residents were asked if problems with ticks had changed over time. The survey was approved by the University of Glasgow College of Medical, Veterinary & Life Sciences Ethics Committee (reference no. 200170121). The survey was available online and in paper copy during April 18–October 31, 2018, and was publicized in local media and at community meetings.

### Statistical Analysis

We conducted statistical analyses and model selection in R version 4.0.0 (https://www.r-project.org) using the lme4 package for generalized linear mixed models (GLMMs) (*37*). We tested for correlations between explanatory variables using the variance inflation function in the car package (*38*). We tested each model for overdispersion. Starting from the maximum global model, we conducted stepwise model selection using likelihood-ratio tests (*39*).

Because Lyme disease incidence is reported at the island level (*6*), we assessed the relationship with the environmental hazard using a 2-step process. First, we investigated island as a predictor of nymph density, nymph infection prevalence, and the density of infected nymphs. Then, we made between-island comparisons from the best fit model using the Tukey test in the lsmeans package (*40*). We modeled nymph abundance (i.e., number of nymphs/10 m transect) from sites sampled in 2018 using a Poisson GLMM with a log link as a function of island, habitat type and wind (using the Beaufort wind force scale), vegetation density, temperature, and humidity with random effects of site and observation (*41*). We modeled the proportion of nymphs infected with *B. burgdorferi* s.l. from sites sampled in 2018 and 2019 using a binomial GLMM with a logit link as a function of island, habitat type, and mean nymph density with a random effect of site. We modeled the density of infected nymphs as the number of infected nymphs using a Poisson GLMM with a log link as a function of island and habitat, with an offset of the log estimated area to collect nymphs tested, using a random effect of site.

For high-incidence islands, where we had sampled additional habitat types, we used separate GLMM models to test for the effect of habitat and island on nymph density, nymph infection prevalence, and the density of infected nymphs. We did not include machair in the analyses because of the low number of nymphs detected.

We used survey responses to test for differences in human exposure to tick bites among islands with high and low Lyme disease incidence. We received 522 surveys from adult residents of the Western Isles, representing approximately 2% of the adult population. According to local census data, survey responses were broadly representative of island populations (Appendix). We modeled risk for tick bite exposure, classified as high (>5 tick bites/year) or low (<5 tick bites/year), using univariable analysis (Appendix Table 1) and then with a binomial GLM and a logit link as a function of island of residence, age, sex, frequency of outdoor activity, and pet ownership. Because awareness, attitudes and preventative behavior relating to tickborne disease could influence reported tick bite exposure, we tested for associations between risk for tick bite exposure and these explanatory variables in a separate model with an interaction of each variable with Lyme disease incidence.

Survey respondents commonly reported ticks in the home; we hypothesized that ticks could be transported indoors by clothing or pets and that this kind of exposure could vary among islands. To test this hypothesis, we used a binomial GLM and a logit link to model whether any tick (live and unfed, engorged, or dead) had ever been detected inside the home as a function of island, level of outdoor activity, and pet ownership.

We hypothesized that a higher proportion of respondents from high-incidence islands would report increasing tick numbers and associated problems than respondents from low Lyme disease incidence islands. We categorized free text responses as increased or not increased and used a binomial GLM with a logit link using Lyme disease incidence as an explanatory variable. We compared free text responses among residents of high- and low-incidence islands to assess factors associated with problems related to ticks. We used a corpus linguistic approach to extract common keywords and associated clusters of words for comparison (*42*; Appendix)

## Results

### Nymph Density

**Figure 2:**
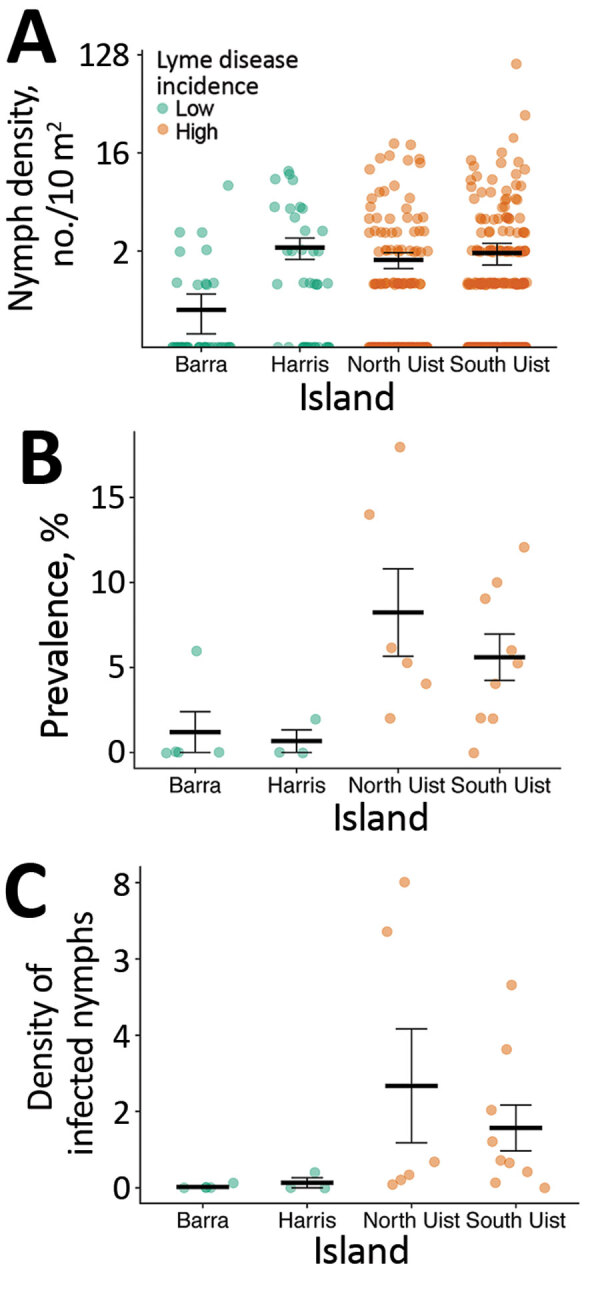
Comparison of nymph density, infection prevalence, and density of infected nymphs by island, Western Isles, Scotland, UK, 2018–2019. A) Nymph density shown by 10 m^2^ blanket drag. B) Prevalence of *Borrelia burgdorferi* sensu lato shown by site. C) Density of infected nymphs per 100 m^2^ shown by site. Green indicates islands with low incidence of Lyme disease; brown indicates islands with high incidence. Data shown from grassland and moorland sites shown in Figure 1. Horizontal bars indicate means and SEs.

Nymph density did not vary significantly between islands with high and low Lyme disease incidence; island was not retained as an explanatory variable in the best fit model (Table 1; χ^2^ = 3.15; degree of freedom [df] = 4; p = 0.53) (Figure 2). In 2018, mean nymph density at improved grassland and heather moorland sites on low Lyme disease incidence islands was 1.36 nymphs/10 m^2^ (SE = 0.28) compared to 1.60 nymphs/10 m^2^ (SE = 0.25) on high-incidence islands (Figure 1; Appendix Table 2).

**Table 1:** Best fit generalized linear mixed models of nymph density, *Borrelia burgdorferi* sensu lato prevalence, and density of infected nymphs among questing *Ixodes ricinus* ticks, Western Isles, Scotland, UK, 2018–2019

Response variable	Explanatory variable	Estimate	SE	p value*
Nymph density	Intercept	−4.02	0.93	
	Temperature, °C/10	2.11	0.64	<0.01
Nymph infection prevalence	Intercept	−2.94	0.30	
	Island			<0.01
	South Uist	Referent		
	North Uist	0.37	0.44	
	Harris	–2.69	1.11	
	Barra	–1.98	0.71	
Density of infected nymphs	Intercept	−4.82	0.52	
	Island			<0.01
	South Uist	Referent		
	North Uist	–0.07	0.79	
	Harris	–2.96	1.45	
	Barra	–4.07	1.15	

*p value determined from likelihood-ratio test of removing each variable from the best fit model.

**Figure 3:**
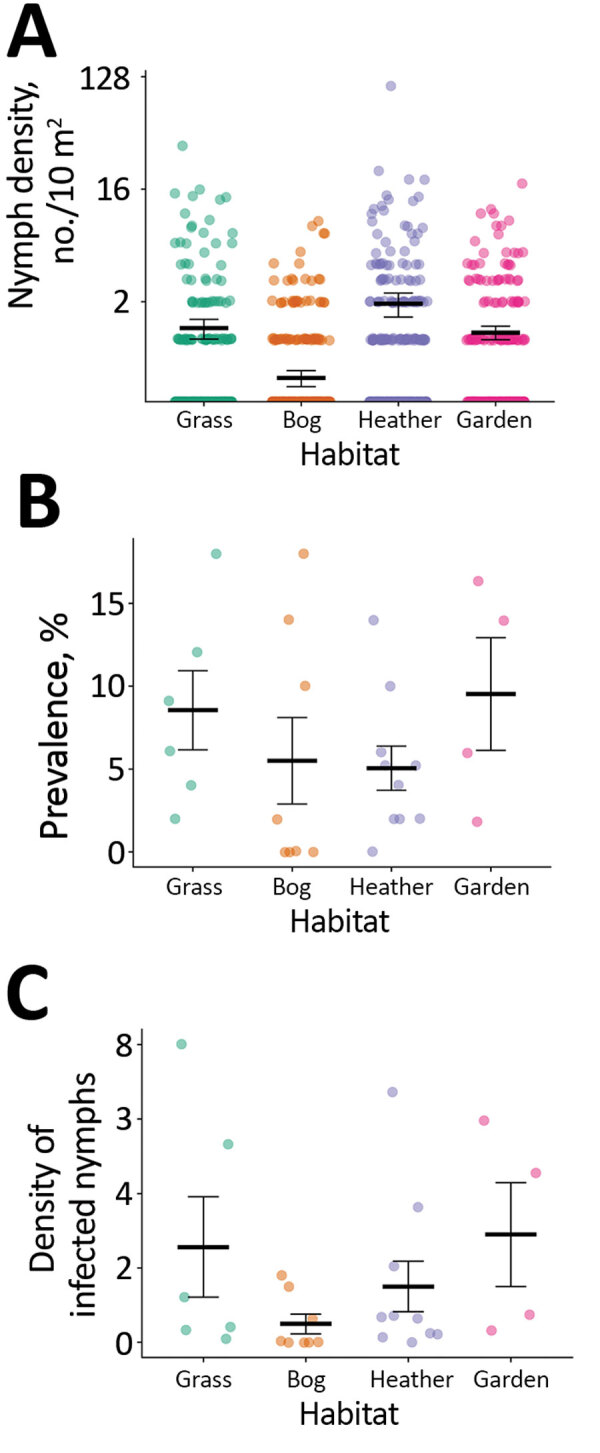
Comparison of nymph density, infection prevalence, and density of infected nymphs by habitat type in islands with high incidence of Lyme disease, Western Isles, Scotland, UK, 2018**.** A) Nymph density shown by 10 m^2^ blanket drag. B) Prevalence of *Borrelia burgdorferi* sensu lato shown by site. C) Density of infected nymphs per 100 m^2^ shown by site. Machair sites not shown because of low mean tick density (0.025 nymphs/10 m^2^; SE = 0.015). Horizontal bars indicate means and SEs.

For sites sampled among different habitat types on high Lyme disease incidence islands (Appendix Figure 1), the best fit model to predict nymph density retained habitat type as a fixed effect (χ^2^ = 24.06; df = 4; p<0.01) (Figure 3; Appendix Table 3). We found significantly fewer nymphs in machair than in other habitat types (p<0.01 by Tukey post hoc analysis); we found no significant differences in nymph density between other habitat types.

### *B. burgdorferi* s.l. Nymph Infection Prevalence

We found that the prevalence of *B. burgdorferi* s.l. infection was significantly associated with island (Table 1; χ^2^ = 17.04; df = 3; p<0.01) (Figure 2). In total, 3 of 4 between-island comparisons showed that prevalence was significantly higher on high-incidence than low-incidence islands. We found no significant differences in prevalence between islands with the same level of Lyme disease incidence (Appendix Table 4).

The mean infection prevalence on high-incidence islands (6.43%; 57/886; SE = 0.82) was higher than on low-incidence islands (0.66%; 4/609; SE = 0.33) (Appendix Table 2). Among sites on high-incidence islands, 98.25% (56/57) of infected nymphs carried *B. afzelii* and 1.75% (1/57) carried *B. garinii*. Among sites on low-incidence islands, 75% (3/4) of infected nymphs carried *B. garinii* and 25% (1/4) carried *B. valaisiana*. Among sites on high-incidence islands, prevalence did not differ by island or habitat type (Appendix Table 3).

### Density of Infected Nymphs

Variation in the density of infected nymphs was significantly associated with island (Table 1; χ^2^ = 16.98; df = 3; p<0.01) (Figure 2). In 2 of 4 between-island comparisons, the density of infected nymphs was significantly higher on high-incidence than low-incidence islands. We found no significant differences between islands with the same level of Lyme disease incidence (Appendix Table 4).

The mean density of infected nymphs was 1.90 nymphs/100 m^2^ (SE = 0.65) on high Lyme disease incidence islands, compared with 0.07 infected nymphs/100 m^2^ (SE = 0.05) on low-incidence islands. Among sites on high-incidence islands, the density of infected nymphs did not differ by island or habitat type (Appendix Table 3).

### Geographic Locations of Tick Bite Risk

Most (64.4%; 333/517) participants provided information on their island of residence and the habitat where their most recent tick bite had occurred (Appendix). In addition, 51.7% (172/333) of these participants also provided the location of their most recent tick bite. Of these bites, 72.7% (125/172) occurred within 1 km of the participant’s home address, including 81 (47.1%) at the home address (Appendix Figure 2).

### Factors Associated with Tick Bite Exposure Risk

In a multivariable model, the most significant explanatory variable for tick bite exposure risk was island of residence (χ^2^ = 20.86; df = 4; p<0.01) (Table 2). Persons >60 years of age had an increased risk for tick bite exposure (odds ratio [OR] 3.88, 95% CI 1.50–11.48). Persons who participated in outdoor activity most days also had an increased risk for tick bite exposure (OR 1.94, 95% CI 1.12–3.49). Residents of high Lyme disease incidence islands had significantly higher rates of tick bite exposure than those of low Lyme disease incidence islands (OR 2.41, 95% CI 1.55–3.82; Appendix Table 1). Awareness, attitudes, and preventative behaviors did not significantly differ between residents living on islands of high and low Lyme disease incidence.

**Table 2:** Best fit general linear model of factors affecting risk for tick bite exposure in residents of the Western Isles, Scotland, UK, 2018–2019

Variable	Estimate	SE	p value*	Odds ratio (95% CI)
Intercept	–1.99	0.54	NA	NA
Island			<0.01	
South Uist	Referent			
North Uist	0.11	0.31		1.12 (0.61–2.07)
Benbecula	–0.85	0.48		0.43 (0.16–1.05)
Barra	0.01	0.42		1.01 (0.43–2.30)
Harris/Lewis	–1.14	0.33		0.32 (0.16–0.61)
Age, y			0.01	
18–30	Referent			
30–60	0.76	0.48		2.14 (0.90–5.97)
>60	1.36	0.51		3.88 (1.50–11.48)
Outdoor activity			0.02	
Less than most days	Referent			
Most days	0.66	0.29		1.94 (1.12–3.49)

*p value determined from likelihood ratio tests of removing each variable from the best-fit model.

### Factors Associated with Finding a Tick within the Home

The chances of finding a tick within the home increased with pet ownership (OR 4.07, 95% CI 2.61–6.41). Persons who participated in outdoor activity most days also had a slightly increased risk (OR 1.67, 1.05–2.64). The likelihood of finding a tick in the home did not vary among islands (Appendix Table 5).

### Changes in Tick Numbers and Problems Over Time

Approximately half (50.6%; 210/415) of respondents described an increase in tick-associated problems over time. Residents from high Lyme disease incidence islands were significantly more likely to report that tick numbers and associated problems had increased over time (OR 4.5, 95% CI 2.1–10.0) (χ^2^ = 15.48; df = 1; p<0.01) (Appendix Table 6). Linguistic analysis of free text comments revealed differences in themes between high and low Lyme disease incidence islands. Residents throughout the surveyed area reported an increased tick presence; residents of high Lyme disease incidence islands were more likely to describe the increase with words such as definitely or significantly than residents of low Lyme disease incidence islands. Residents of high Lyme disease incidence islands were also more likely to report deer near their homes (Appendix Table 7).

## Discussion

We investigated Lyme disease emergence in treeless habitats in Europe. Our findings show that environmental hazard and human tick bite exposure risk contribute to higher Lyme disease incidence in these settings. In contrast to previous studies in Europe, we found that the density of infected nymphs in treeless habitats can be comparable to forested sites, which are traditionally associated with higher Lyme disease hazard (*34*,*43*).

We found a significantly higher prevalence of *B. burgdorferi* s.l. infected nymphs among high Lyme disease incidence islands, which contributed to a higher environmental hazard on these islands. Almost all infected ticks on these islands carried *B. afzelii,* a genospecies associated with mammalian transmission hosts (*44*). We did not detect *B. afzelii* infection in ticks collected from low Lyme disease incidence islands, where the prevalence of infection in ticks was extremely low (<1%). Because of the similarity in habitats and climate, we hypothesize that the presence or absence of this genospecies could be driven by differences in the host community. Alternatively, the introduction of *B. afzelii* from the mainland might have been limited to certain islands.

Within islands with a high incidence of Lyme disease, we found that improved grassland, heather moorland, bog and peatland, and domestic gardens had similar tick density and prevalence of *B. burgdorferi* s.l. infection among ticks as forested mainland sites in Scotland (*34*,*43*). Our results suggest that microclimatic conditions in these open habitats, possibly driven by the milder oceanic climate on the Western Isles, can be as conducive to tick survival as conditions in woodlands. Tick abundance was positively associated with vegetation density, which when combined with relatively high rainfall and humidity in this location, might contribute to a favorable microclimate and improved off-host tick survival. In contrast, we found significantly lower tick abundance within machair grassland, probably caused by a combination of short vegetation height, lack of a vegetation mat, and agricultural rotations and ploughing, which can reduce off-host tick survival (*45*,*46*). Tick abundance varied considerably within habitats (Appendix Table 2), a finding that warrants further investigation.

In addition to a higher environmental hazard on high Lyme disease incidence islands, residents of these islands reported more frequent exposures to tick bites. Tick bite exposure increased with the participant’s age and amount of outdoor activity. Although outdoor activity and knowledge, attitudes, and prevention of tick bites did not contribute to differences in tick bite exposure between islands with high and low Lyme disease incidence, this finding might have been affected by the higher proportion of responses from older residents on high Lyme disease incidence islands. Although we found no significant differences in tick density between high- and low-incidence grassland and moorland sites, survey responses indicated that most tick bites occurred close to the home address, and frequently in gardens. On high Lyme disease incidence islands, we found a similar density of infected nymphs in gardens to surrounding habitats, indicating that spillover of infected ticks is common. Further research is required to test whether peridomestic tick exposure contributes to differences in tick bite exposure between islands. The findings that tick bites frequently occur within gardens and that residents might be exposed to ticks within their homes suggest that all members of a household could be at risk for tick bites. Our research suggests that environmental and educational public health interventions focused around residences could reduce tick bite exposure and potentially cases of Lyme disease.

Similar to previous studies, we found that in the absence of longitudinal data on vector populations and linked ecologic drivers, community surveys can be valuable indicators of ecologic trends (*31*). Residents of high Lyme disease incidence islands were significantly more likely to report that ticks were an increasing problem. In addition, many of these participants suggested that increased deer populations and presence near homes might contribute to increased numbers of ticks. Because deer habitat use and tick density are established drivers of tick populations and distribution (*27*,*47*,*48*) and are associated with Lyme disease emergence in other areas of Europe (*49*), this association should be investigated in future research.

In summary, we have shown that treeless habitats can support similar tick densities and infection risk as forested areas and can be associated with Lyme disease emergence in humans. Our results suggest the potential for Lyme disease to emerge in open habitats with a suitable microclimate for off-host tick survival and host availability for blood meals elsewhere in Europe. Integrating these results with human data on human exposure to tick bites revealed that most tick bites occurred close to homes. Furthermore, we found that the spillover of ticks and tickborne pathogens into gardens and homes is an emerging problem that residents attribute to increased deer populations and their changing distribution. Further research to understand the effects of ecologic drivers of tick populations in these regions, together with information on human use of these environments, is necessary to achieve more accurate prediction of areas of risk and suggest ways to prevent and mitigate this risk.

Appendix Further information on emergence of Lyme disease on treeless islands, Scotland, UK.
