# Supplementary material for: Emergence of Lyme Disease on Treeless Islands, Scotland, United Kingdom
Source: Emerg Infect Dis. 2021 Feb;27(2):538–46. doi: 10.3201/eid2702.203862 (PMC7853560; doi:10.3201/eid2702.203862)
Supplement: Appendix — Further information on emergence of Lyme disease on treeless islands, Scotland, UK. [file 20-3862-Techapp-s1.pdf]

Article DOI: https://doi.org/10.3201/eid2702.203862

Emergence of Lyme Disease on Treeless Islands, Scotland, United Kingdom

Appendix

Corpus Linguistic Approach to Extract Common Keywords from Free Text

Corpus linguistic methods were used to explore the meanings in free-text responses of respondents to the question: "Do you think tick numbers and problems with ticks have changed over time?" The text responses were imported from MS Excel (Microsoft, https://www.microsoft.com) into notepad to form a corpus and then imported into WordSmith Tools version 7 (Lexical Analysis Software Ltd., https://lexically.net/wordsmith), a corpus linguistic analytical software tool. Corpus linguistic analysis was performed as previously described (*1*).

The free-text responses were first analyzed across all islands and then separated by high- or low- Lyme disease incidence island group. Before analysis, the words contained within each question were included in a "stop list" in the analysis for each question, which removed suggestion bias from the responses and prevented those words from being identified as keywords.

Keyword analysis identified keywords in the grouped responses for each question. Each keyword had an associated key-ness value on the basis of comparison to the frequency of that word in the British National Corpus. The log-likelihood value was used as a measure of key-ness as per standard option in Wordsmith Tools (*2*). Keyword clusters also were presented to show the meanings within each text.

Further exploration of the main keywords using concordance and collocation analysis enabled further exploration of the context around the main keywords. In other words, this contextual analysis revealed additional meanings in survey responses.

Comparison of Survey Responses with Island Demographics

We compared the survey participants' age and sex to census data from the Western Isles (*3*). We calculated the proportion of responses from each age group and from high- and low-Lyme disease incidence islands. There was good representation from different age groups; 1.5% of residents 18–30 years of age, 2.9% of residents 30–60 years of age, and 1.1% of the >60 years of age responded to the survey. Relatively more responses were received from high-incidence islands (5% of the population) compared with low-incidence islands (approximately 1% of the population). A greater proportion of survey responses were from women (69%) than men (31%).

Habitat Types of Reported Human Tick Bites

Most (333/517; 64.4%) participants provided information on the habitat type of their last tick bite and island of residence. Most tick bites occurred in heather moorland (131/333; 39.3%), improved grassland (92/333; 27.6%), gardens (88/333; 26.4%), and machair grassland (22/333; 6.6%). In high Lyme disease incidence–areas, tick bites tended to occur more often in gardens and fewer occurred in machair grassland (gardens: p = 0.05 by X^2 test; 26/127 low Lyme disease incidence–islands vs. 62/206 high Lyme disease incidence–islands) (machair grassland: p = 0.04 by X^2 test; 13/127 low Lyme disease incidence–islands vs. 9/206 high Lyme disease incidence–islands).

References

1. Huntley SJ, Mahlberg M, Wiegand V, van Gennip Y, Yang H, Dean RS, et al. Analysing the opinions of UK veterinarians on practice-based research using corpus linguistic and mathematical methods. Prev Vet Med. 2018;150:60–9. PubMed https://doi.org/10.1016/j.prevetmed.2017.11.020

2. Dunning T. Accurate methods for the statistics of surprise and coincidence. Comput Linguist Assoc Comput Linguist. 1993;19:61–74.

3. Comhairle nan Eilean Siar. 2011 census. 2011 [cited 2020 Oct 20]. https://www.cne-siar.gov.uk/strategy-performance-and-research/outer-hebrides-factfile/population/2011-census

4. National Health Service—Western Isles. The 'tick'-ing time bomb: the incidence of Lyme disease in the Outer Hebrides (2010–2017). 2018 [cited 2020 Aug 13]. https://www.wihb.scot.nhs.uk/wp-content/uploads/2020/08/A0-Template-The-ticking-time-bomb.-Incidence-of-Lyme-disease-in-the-Western-Isles-2010-2017.pdf

Appendix Table 1. Univariable analysis of risk factors for tick bites reported in surveys of residents of the Western Isles (classified as high, ≥5 tick bites a year, or low, < 5 tick bites a year), Western Isles, Scotland, United Kingdom, 2018

Variable	Responses	Sample size*	Odds ratio (95% CI)	p value†
Age, y	458			
18–30		8/46	Referent	<0.01
30–60		77/321	1.50 (0.70–1.60)	
>60		35/91	2.99 (1.30–7.52)	
Sex	460			
F		85/318	Referent	0.88
M		37/142	0.97 (0.61–1.51)	
Island	455			
South Uist		41/114	Referent	<0.01
North Uist		38/102	1.06 (0.61–1.84)	
Benbecula		8/43	0.41 (0.16–0.92)	
Barra		13/40	0.86 (0.39–1.82)	
Harris, Lewis		21/156	0.28 (0.15–0.50)	
Lyme disease incidence‡	455			
Low		34/196	Referent	<0.01
High		87/259	2.41 (1.55–3.82)	
Occupation	437			
Indoor		77/331	Referent	<0.01
Outdoor		15/48	1.50 (0.76–2.86)	
Retired		27/58	2.87 (1.61–5.11)	
Outdoor activity	432			
<Most days		21/120	Referent	0.01
Most days		91/312	1.94 (1.16–3.37)	
Cat/dog ownership	460			
No		37/129	Referent	0.51
Yes		85/331	0.86 (0.55–1.36)	
Accessed information about ticks or Lyme disease	453			
No		7/34	Referent	0.39
Yes		114/419	1.44 (0.64–3.68)	
Perception of risk from tick bites	453			
Minor		23/137	Referent	<0.01
Substantial		55/196	1.93 (1.12–3.39)	
Serious		44/120	2.87 (1.62–5.20)	
Prevention measures used	296			
None		15/68	Referent	<0.01
Special clothing		47/135	1.89 (0.98–3.80)	
Deer fence+/–other		17/27	6.01(2.33–16.38)	
Other		12/66	0.78 (0.33–1.83)	
Frequency of checking for tick bites**, %**	449			
<10		17/179	Referent	<0.01
11–50		18/78	2.86 (1.38–5.95)	
51–99		53/112	8.56 (4.68–16.34)	
100		34/80	7.04 (3.66–14.01)	

*Data expressed as no. respondents with high tick bite exposure (≥5 tick bites a year)/no. respondents.
†p value determined from likelihood-ratio test compared to a null model.
‡Lyme disease incidence classified as high (North Uist, South Uist, and Benbecula) or low (Barra, Harris, and Lewis) (*4*).

Appendix Table 2. *Ixodes ricinus* nymph density and *Borrelia burgdorferi* sensu lato infection prevalence in study sites, Western Isles, Scotland, United Kingdom, 2018–2019*

Lyme disease incidence†	Island	Habitat	Year	Coordinates	Total nymphs collected	Nymphs per 100 m	Nymphs tested	Prevalence of *B. burgdorferi* sensu lato, %
Low	South Harris	Improved grassland	2018	57.82297°N, –7.04121°E	100	32.5	100	0
			2019	57.86033°N, –6.76844°E	190	20	50	2
			2019	57.85990°N, –6.97867°E	1	0	NA	NA
			2019	57.83909°N, –6.75505°E	6	0	NA	NA
		Heather moorland	2018	57.76642°N, -6.99558°E	100	10.5	100	0
			2019	57.85464°N, –6.77910°E	9	1	NA	NA
			2019	57.85119°N, –6.96178°E	21	1	NA	NA
			2019	57.81542°N, –6.92514°E	1	0	NA	NA
	Barra	Improved grassland	2018	57.00991°N, –7.49054°E	98	5.5	98	0
			2019	56.98296°N, –7.50262°E	1	0.5	NA	NA
			2019	56.99873°N, –7.49991°E	1	0	NA	NA
			2019	57.04221°N, –7.42730°E	1	0	NA	NA
		Heather moorland	2018	57.01508°N, –7.45006°E	110	6	100	0
			2019	56.96077°N, –7.51683°E	59	4.5	54	0
			2019	56.97012°N, –7.50559°E	137	8.5	57	0
			2019	56.97535°N, –7.42723°E	84	2	50	6
High	North Uist	Improved grassland	2018	57.64579°N, –7.27850°E	18	1	NA	NA
			2018	57.59542°N, –7.37803°E	59	44.5	50	18
			2018	57.55027°N, –7.27865°E	51	17	50	2
			2018	57.55679°N, –7.36161°E	52	1.5	49	6.12
		Heather moorland	2018	57.64992°N, –7.47042°E	52	17	50	4
			2018	57.56901°N, –7.28658°E	15	0	NA	NA
			2018	57.57699°N, –7.35361°E	57	48	50	14
			2018	57.62612°N, –7.20569°E	76	4	76	5.26
	Benbecula	Improved grassland	2018	57.41507°N, –7.30903°E	9	1	NA	NA
			2018	57.42847°N, –7.35645°E	23	2.5	NA	NA
		Heather moorland	2018	57.43784°N, –7.36701°E	51	12	50	2
			2018	57.46292°N, –7.29770°E	20	2	NA	NA
	South Uist	Improved grassland	2018	57.39698°N, –7.34315°E	9	0.5	NA	NA
			2018	57.33157°N, –7.36658°E	0	0	NA	NA
			2018	57.27389°N, –7.39276°E	76	10	58	12.07
			2018	57.19942°N, –7.40313°E	76	10.5	50	4

Lyme disease incidence†	Island	Habitat	Year	Coordinates	Total nymphs collected	Nymphs per 100 m	Nymphs tested	Prevalence of *B. burgdorferi* sensu lato, %
High	South Uist	Improved grassland	2018	57.16089°N, –7.30559°E	251	58.5	77	9.09
			2018	57.12438°N, –7.37993°E	35	2	NA	NA
		Heather moorland	2018	57.30218°N, –7.35176°E	53	34	50	6
			2018	57.23865°N, –7.32935°E	50	31.5	50	0
			2018	57.23901°N, –7.36996°E	55	6.5	50	10
			2018	57.13368°N, –7.34022°E	50	7	50	2
			2018	57.26320°N, –7.27952°E	50	36	50	2
			2018	57.33028°N, –7.30772°E	138	69	76	5.26
			2018	57.33718°N, –7.35609°E	25	2.5	NA	NA
			2018	57.13750°N, –7.29402°E	9	0.5	NA	NA
	North Uist	Bog, peatland	2018	57.61533°N, –7.20634°E	51	1.5	50	2
			2018	57.64040°N, –7.42523°E	56	4.5	50	14
			2018	57.57971°N, –7.24579°E	21	1.5	NA	NA
			2018	57.53646°N, –7.31195°E	50	15	50	0
		Garden‡	2018	sector 2	13	6.5	NA	NA
			2018	sector 3	11	5.5	NA	NA
			2018	sector 4	20	10	NA	NA
			2018	sector 5	2	1	NA	NA
		Machair	2018	57.66923°N, –7.24728°E	0	0	NA	NA
			2018	57.59891°N, –7.52762°E	0	0	NA	NA
			2018	57.57246°N, –7.47268°E	0	0	NA	NA
	Benbecula	Bog, peatland	2018	57.46696°N, –7.33463°E	18	1	NA	NA
		Garden	2018	sector 7	11	5.5	NA	NA
		Machair	2018	57.42549°N, –7.37725°E	0	0	NA	NA
	South Uist	Bog, peatland	2018	57.32618°N, –7.27926°E	50	4.5	50	0
			2018	57.24386°N, –7.32181°E	59	10	50	18
			2018	57.12952°N, –7.30258°E	36	2.5	NA	NA
			2018	57.15575°N, –7.37453°E	50	2.5	50	0
			2018	57.34627°N, –7.26833°E	76	8.5	50	0
			2018	57.24486°N, –7.35349°E	270	15	50	10
			2018	57.34174°N, –7.34557°E	32	1.5	NA	NA
			2018	57.27817°N, –7.37005°E	4	0	NA	NA
		Garden	2018	sector 8	3	1.5	NA	NA
			2018	sector 9	64	12.5	50	6
			2018	sector 11	193	32.5	50	14
			2018	sector 12	100	17.5	56	1.79
			2018	sector 13	16	4	NA	NA
			2018	sector 14	73	36.5	49	16.33
			2018	sector 15	6	2.5	NA	NA

Lyme disease incidence†	Island	Habitat	Year	Coordinates	Total nymphs collected	Nymphs per 100 m	Nymphs tested	Prevalence of *B. burgdorferi* sensu lato, %
High	South Uist	Machair	2018	57.35096°N, –7.39092°E	2	1	NA	NA
			2018	57.30452°N, –7.39269°E	0	0	NA	NA
			2018	57.24395°N, –7.42612°E	6	1	NA	NA
			2018	57.15629°N, –7.40349°E	0	0	NA	NA

*NA, not applicable.
†Lyme disease incidence classified as high (North Uist, South Uist, and Benbecula) or low (Barra, Harris, and Lewis) (*4*).
‡Gardens were defined as areas next to a dwelling that were enclosed by a fence to restrict entry of livestock but not deer. Gardens ranged from 0.11–0.21 ha. Gardens typically had a mowed lawn, with areas of shrubs, longer grass and trees. Latitude and longitude are not given for privacy reasons.

Appendix Table 3. Best fit generalized linear mixed models of questing nymph density, *Borrelia burgdorferi* prevalence, and density of infected nymphs among *Ixodes ricinus* ticks from different habitats on islands with high Lyme disease incidence, Western Isles, Scotland, United Kingdom, 2018*

Response variable	Explanatory variable	Estimate	Standard error	p value†
Nymph density	(Intercept)	0.14	0.75	NA
	Habitat type			
	Heather moorland	Referent		
	Improved grassland	–0.34	0.51	<0.01
	Bog and peatland	–0.76	0.49	
	Machair	–3.32	0.80	
	Garden	0.17	0.51	
	Vegetation density	0.14	0.04	<0.01
	Humidity	−0.02	0.01	0.02
Nymph infection prevalence (island)	(Intercept)	−3.01	0.25	NA
Nymph infection prevalence (habitat)	(Intercept)	−2.97	0.28	NA
Density of infected nymphs (island)	(Intercept)	−5.52	0.42	NA
Density of infected nymphs (habitat)	(Intercept)	−5.13	0.43	NA

*Machair sites were excluded because of low nymph density at all sampled sites. Garden sites were excluded to test for differences between islands; North Uist did not have the minimum sample size of ticks collected from individual gardens. To test for differences among islands within high LD incidence areas, and habitat types excluding gardens and machair, 23 sites on North and South Uist, and grassland, moorland and bog and peatland sites were included. To test for differences in prevalence between gardens and other habitats, 18 sites on South Uist from garden, grassland, moorland and bog and peatland sites were included.
†p value determined from likelihood-ratio test compared to removing the variable from the best fit model.

Appendix Table 4. Between-island comparisons of nymph infection prevalence and the density of infected nymphs, from the best fit models shown in Table 1 in the main text, Western Isles, Scotland, United Kingdom, 2018–2019*

Outcome variable	Between-island comparison	Lyme disease incidence comparison	Estimate	Standard error	p value
Nymph infection prevalence					
	South Uist vs. Harris	High vs. low	2.69	1.11	0.07
	South Uist vs. Barra	High vs. low	1.98	0.71	0.03
	North Uist vs. Harris	High vs. low	3.07	1.12	0.03
	North Uist vs. Barra	High vs. low	2.35	0.73	0.01
	Harris vs. Barra	Low vs. low	–0.71	1.25	0.94
	South Uist vs. North Uist	High vs. high	–0.37	0.44	0.83
Density of infected nymphs					
	South Uist vs. Harris	High vs. low	2.96	1.45	0.17
	South Uist vs. Barra	High vs. low	4.07	1.15	<0.01
	North Uist vs. Harris	High vs. low	2.89	1.48	0.21
	North Uist vs. Barra	High vs. low	4.00	1.20	<0.01
	Harris vs. Barra	Low vs. low	1.10	1.68	0.91
	South Uist vs. North Uist	High vs. high	0.07	0.79	1.00

*Between-island comparisons made using Tukey's post hoc test.

Appendix Table 5. Results of best-fit general linear model to assess risk factors for tick detection within survey respondents' homes, Western Isles, Scotland, United Kingdom, 2018*

Variable	Estimate	Standard error	p value†	Odds ratio (95% CI)
Intercept	–0.72	0.24	NA	NA
Cat/dog ownership				
No	Referent			
Yes	1.4	0.23	<0.01	4.07 (2.61–6.41)
Outdoor activity				
Less than most days	Referent			
Most days	0.51	0.23	0.03	1.67 (1.05–2.64)

*Tick presence in a respondents' home was reported by 274/424 (63.7%) respondents who answered all survey questions. The presence of live unfed ticks, which pose a biting risk to humans, was reported by 120/424 (28.3%) respondents. NA, not applicable.
†p value determined from likelihood-ratio test compared to removing the variable from the best fit model.

Appendix Table 6. General linear model of perceived increase in tick numbers and associated problems over time, Western Isles, Scotland, United Kingdom, 2018

Variable	Estimate	Standard error	p value*	Odds ratio (95% CI)
Intercept	1.07	0.24	NA	NA
Lyme disease incidence†				
Low	Referent			
High	1.5	0.4	<0.01	4.46 (2.10–10.02)

*p value determined from likelihood-ratio test compared to removing the variable from the best fit model.
†Lyme disease incidence classified as high (North Uist, South Uist, and Benbecula) or low (Barra, Harris, and Lewis) (*4*).

Appendix Table 7. Comparison of collocated words in response to survey question "Do you think tick numbers and problems with ticks have changed over time?" Western Isles, Scotland, United Kingdom, 2018

Keyword	Overall position (Log likelihood)	Lyme disease incidence	Collocated words	Collocated clusters (no.)
Deer	1 (601)	High	More, about, ticks, numbers, garden, close	More deer (7), Deer and (5), the deer (5), of deer (4), deer about (3), And Ticks (3), So deer (3)
		Low	Sheep, ticks, more	The deer (4), deer are (3), on the (3)
Increased	2 (445)	High	Years, numbers, tick(s), last, significantly, definitely	Have increased (12), definitely increased (5), increased significantly (4), increased over (4), increased dramatically (4), dramatically increased (4), moorland increased (3)
		Low	Numbers, more, ticks, sheep	Have increased (14), to have (5), they have (4), seem to (3)
Sheep	3 (272)	High	Dipping, dipped, no	Sheep, dipping (3)
		Low	Deer, dipping, numbers, increased	Sheep dipping (3), the sheep (3), on the (3)
